# Case Report: Prolonged Anorexia With Nausea Caused by Immune Checkpoint Inhibitors for Malignant Melanoma Treated Using Kampo Medicines Bukuryoingohangekobokuto and Ninjin’yoeito

**DOI:** 10.3389/fphar.2022.870823

**Published:** 2022-04-12

**Authors:** Shin Takayama, Ryutaro Arita, Tadashi Ishii

**Affiliations:** Department of Education and Support for Regional Medicine (General Medicine and Kampo Medicine), Tohoku University Hospital, Sendai, Japan

**Keywords:** immune checkpoint inhibitors, side effect, anorexia, recover, kampo medicine

## Abstract

Immune checkpoint inhibitors (ICIs) are indicated for several cancers, including malignant melanoma. Anorexia and nausea resulting in malnutrition are side effects of ICIs. In such cases, conventional drugs are used for symptom relief, but the symptoms may persist. We report a case of advanced malignant melanoma with prolonged anorexia and nausea, which occurred after nivolumab administration, and was successfully treated using Kampo medicines. A 75-year-old man with nasal bleeding visited our hospital. A nasal scope revealed an obstructive tumor in the left nasal concha. Tissue biopsy showed malignant melanoma, and computed tomography showed metastasis to the liver and bone. Thus, the patient was diagnosed with stage IV malignant melanoma. He received radiotherapy (30 Gy) and nivolumab with ipilimumab four times, followed by nivolumab administration alone. During the administration of nivolumab, he complained of severe anorexia and nausea, with a numeric rating scale (no symptoms, 0; severe symptoms, 10) score of 10. He could not consume food because of these symptoms, even after nivolumab administration was discontinued. His blood pressure was 92/59 mmHg, his performance status (PS; no fatigue, 0; bedridden or disabled, 4) was 4, and his body weight gradually decreased from 60 to 39 kg in a month. The patient showed malnutrition and dehydration and experienced anxiety and depression. Nivolumab was terminated, and conventional symptomatic drugs were prescribed, but the symptoms persisted. We then prescribed 9.0 g/day of ninjin’yoeito (TJ-108, Tsumura and Co.) to allow recovery from anorexia and subsequently added bukuryoingohangekobokuto (TJ-116, Tsumura and Co.) to treat the persistent nausea. After treatment with these two Kampo medicines, the patient’s appetite gradually recovered. Along with the recovery of nutritional status, his PS improved to 0, his anxiety and depressive state improved, and his body weight increased to 60 kg. The patient remained in good condition without cancer recurrence. The patient’s clinical course shows the usefulness of Kampo medicine as supportive care for symptom relief and maintenance of nutritional and mental status during cancer treatment.

## Introduction

Several immune checkpoint inhibitors (ICIs) have been developed to treat cancers, including malignant melanoma. Side effects of nivolumab have been reported, including appetite loss and nausea (JAPIC, 2014), with an incidence of >5.0%. Anorexia and nausea, resulting in poor nutritional status, are general problems associated with treatment using ICIs. Conventional drugs for symptom relief are used in these cases; however, symptoms are occasionally refractory to such treatment. Prolonged appetite loss due to nausea causes malnutrition, which leads to frailty, and influences mental status.

In contrast, the use of Kampo medicines for patients with cancer was reported to be >70% among physicians in core cancer treatment hospitals (Ito et al., 2012). The application of Kampo medicines, including ginseng, such as rikkunsito (RKT), has been reported in the treatment of anorexia as a side effect of cancer treatment (Yoshiya et al., 2020). We previously reported long-term survival and improvement in quality of life in patients with advanced cancer, including pancreatic cancer, brain cancer, and esophageal cancer, which was supported with Kampo medicines (Shimizu et al., 2021a; Shimizu et al., 2021b; Suzuki et al., 2021; Takayama and Ishii, 2022). Ninjin’yoeito (NYT) allows recovery from anorexia with frailty, and bukuryoingohangekobokuto (BRGHT) is used for persistent nausea with anxiety.

Kampo medicines is used according to slight and minor symptoms, considering the patient’s body composition, constitution, and characteristics. According to symptoms and physical findings, RKT is used for the treatment of anorexia with qi (energy) deficiency and fluid retention; NYT is used to treat anorexia and malnutrition with qi deficiency and blood deficiency; and BRGHT is used to treat anorexia, nausea, vomiting, and anxiety with qi deficiency, qi counterflow, phlegm, and dampness in the traditional medicine setting.

Here, we report a case of advanced malignant melanoma with severe appetite loss and nausea, which occurred after ICIs administration and was recovered through Kampo medicine.

## Case Description

**FIGURE 1:**
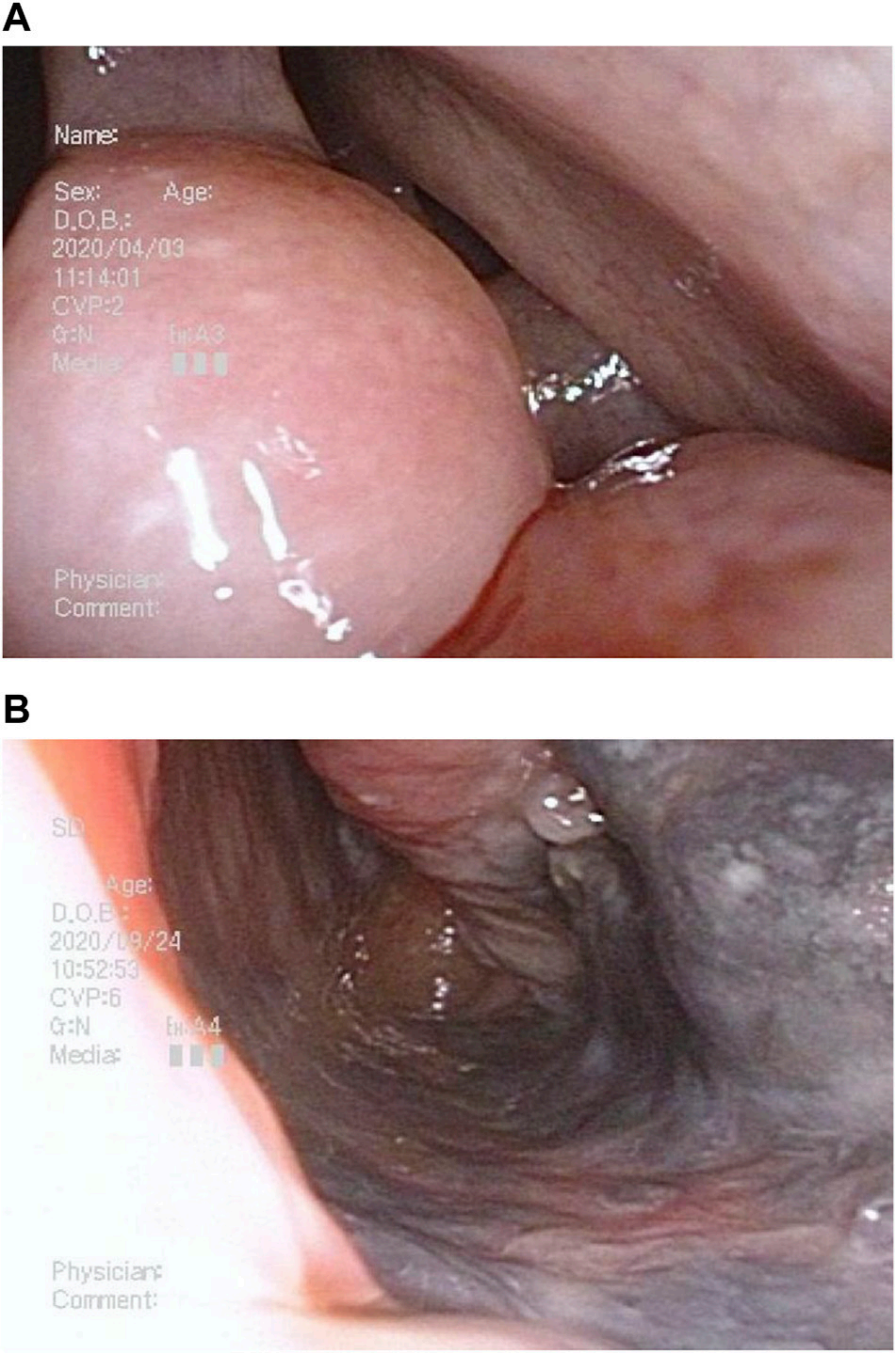
Nasal scope at the first visit and after radiotherapy and chemotherapy. **(A)** First visit: left nasal concha occupied by the tumor. **(B)** Tumor remission after radiotherapy followed by nivolumab and ipilimumab therapy.

**FIGURE 2:**
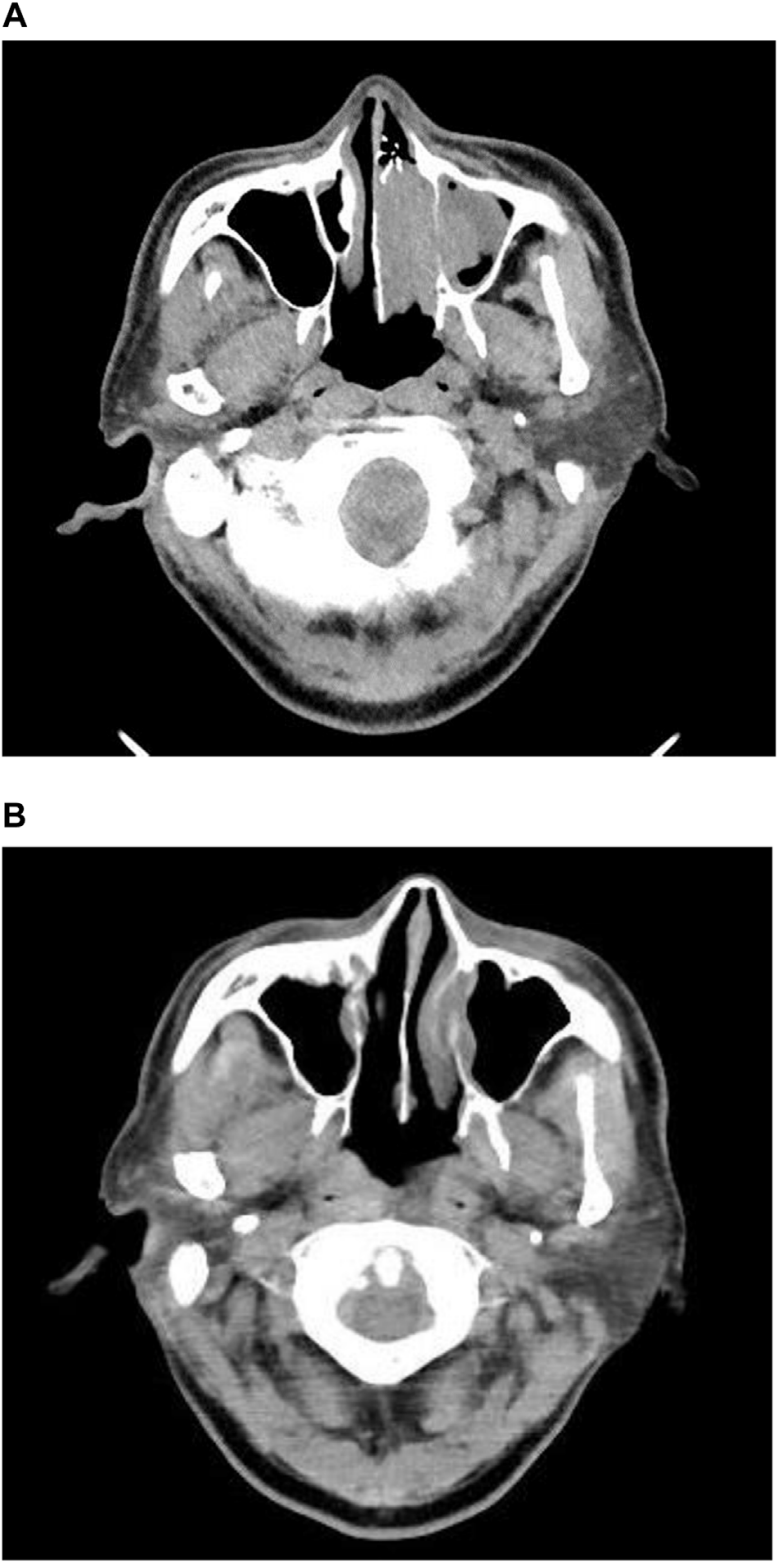
Contrast computed tomography at the first visit and after radiotherapy and chemotherapy **(A)**. First visit: The left nasal concha was obstructed by the tumor. **(B)** Tumor remission after radiotherapy followed by nivolumab and ipilimumab therapy.

A 75-year-old man with a history of myocardial infarction and habituation to smoking complained of nasal bleeding and visited our hospital. A nasal scope revealed an obstructive tumor in the left nasal concha (Figure 1A). Tissue biopsy showed malignant melanoma, and computed tomography (Figure 2A) showed the obstructed tumor at the left nasal concha with metastases in the liver and bone; thus, he was diagnosed with stage IV malignant melanoma. Programmed death-ligand 1 (PD-L1) test using a biopsy showed positive results, suggesting that anti-cancer drugs and ICIs could effectively treat the advanced-stage malignant melanoma. The patient received radiotherapy (30 Gy) and nivolumab with ipilimumab four times, followed by the sole administration of nivolumab. Subsequently, the patient complained of severe anorexia with nausea, with a numerical rating scale (NRS; no symptoms, 0; severe symptoms, 10) score of 10. He also complained of taste disorder. The patient was unable to eat because of these symptoms. At the first visit to our outpatient clinic, his blood pressure was 92/59 mmHg, and his performance status (PS; no fatigue, 0; bedridden or disabling, 4) (Date, 1999) was 4. His body weight gradually decreased from 60 to 39 kg in a month. His body mass index decreased to 15.7, with a blood test showing total protein (TP) 6.0 g/dL, albumin (Alb) 3.2 g/dL, creatinine (Cr) 1.38 mg/dL, and lymphocyte count 1780/μL. The patient was positive for malnutrition and dehydration, and he experienced anxiety and depression. Anorexia, nausea, and renal failure were suspected to be adverse reactions to the ICIs. Hence, nivolumab was terminated; but the symptoms persisted. Conventional drugs, such as mosapride citrate hydrate, metoclopramide, and lansoprazole, were prescribed, but they did not eliminate the symptoms. Despite the tumor reduction (Figures 1B, 2B), his PS, mental status, and quality of life decreased after cancer remission.

**FIGURE 3:**
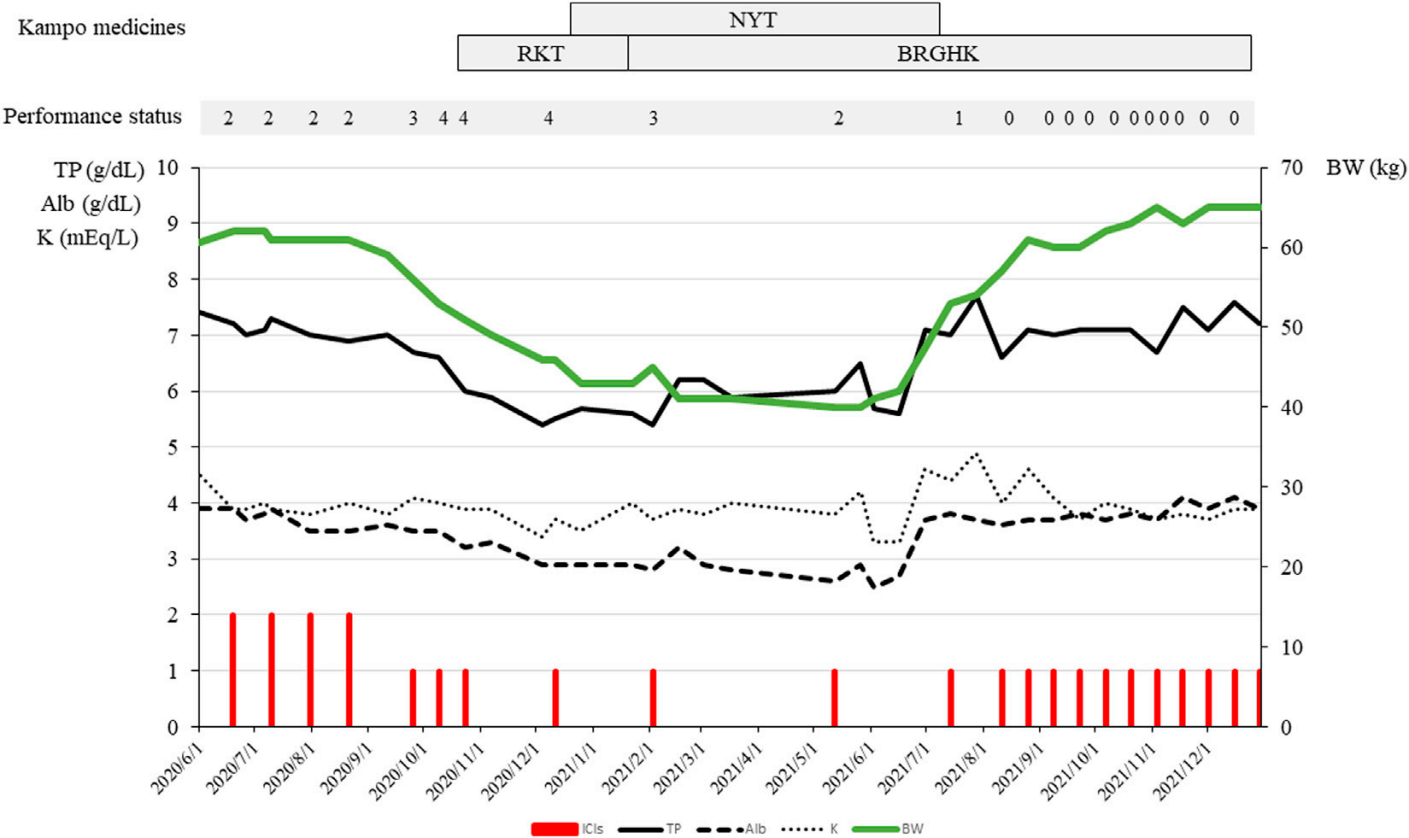
Clinical course of the treatment. Performance status is rated from 0 to 4 for fatigue (lethargy, malaise, and asthenia); 0: none, 1: increased fatigue over baseline, but not altering normal activities; 2: moderate or causing difficulty in performing some activities; 3: severe or loss of ability to perform some activities; 4: bedridden or disabled (Date, 1999).

According to traditional medicine diagnosis, persistent nausea and anorexia, with a weak pulse and hypochondrium stuffiness, showed qi deficiency with fluid retention. We prescribed 7.5 g/day of RKT (TJ-43, Tsumura and Co., see STORK http://mpdb.nibiohn.go.jp/stork/) to alleviate the nausea. Two weeks after this prescription, the NRS score for nausea had decreased from 10 to 7. His anxiety slightly reduced because he could now consume solid food, but the improvement was insufficient. According to traditional medicine diagnosis, residual anorexia and malnutrition with a weak pulse showed qi deficiency and blood deficiency. Thus, we added 9.0 g/day of NYT (TJ-108, Tsumura and Co., see STORK http://mpdb.nibiohn.go.jp/stork/) to allow recovery from anorexia and malnutrition. Because his pulse remained weak and water brash from the stomach to esophagus persisted, indicating qi deficiency, qi counterflow, phlegm, and dampness in the traditional medicine setting, we changed RKT to BRGHT (TJ-116, Tsumura and Co., see STORK http://mpdb.nibiohn.go.jp/stork/) to treat the persistent anorexia, nausea, vomiting, and anxiety. After taking these two Kampo medicines, his appetite gradually recovered, and nutritional status indicators such as TP, Alb, and body weight gradually increased. His PS improved from 4 to 0, and his anxiety and depressive state also improved, with his body weight returning to 60 kg. Figure 3 shows the clinical course of the treatment.

After the re-administration of nivolumab under Kampo treatment, the patient remained in good condition, with no cancer recurrence.

## Discussion

Recently, ICIs, such as human monoclonal anti-human PD-1 antibody, have been developed for several cancers, including malignant melanoma. Anorexia, nausea, and poor nutritional status are side effects of cancer treatment, including the use of ICIs. Prolonged anorexia and nausea cause malnutrition and mental disorders, leading to frailty during cancer treatment. Conventional treatment is used as supportive care to relieve these symptoms, but the symptoms occasionally persist. Delayed or prolonged effects and adverse reactions with ICIs have also been known. Furthermore, severe anorexia might be induced by combining nivolumab and ipilimumab rather than nivolumab alone.

In Japan, physicians can prescribe 148 types of Kampo medicines under the national health insurance system. Hospitalized patients undergoing cancer treatment *via* gastrointestinal and gynecological surgery receive several Kampo medicines (Sugimine et al., 2021). Recently, randomized controlled trials (RCTs) showed the efficacy and safety of Kampo medicines, including in cancer treatment (Motoo et al., 2021).

Frailty and cachexia related to cancer are serious problems. Anamorelin hydrochloride is a ghrelin mimetic agent for the treatment of cancer cachexia. However, its indication is limited to patients with unresectable advanced or recurrent non-small cell lung cancer, gastric cancer, pancreatic cancer, and colorectal cancer. Anamorelin hydrochloride is not indicated in cases of malignant melanoma, such as the present case. Supportive care during cancer treatment, including using Kampo medicine, is occasionally indicated to relieve symptoms such as fatigue, nausea, appetite loss, constipation, diarrhea, and abdominal pain. Table 1 shows the list of Kampo medicines, including ginseng, for symptom relief in cancer treatment.

**TABLE 1:** Amounts of crude drugs in Kampo medicines, RKT, BRGHT, and NYT (top of the table); and symptoms and conditions of application (bottom).

Crude Drugs	RTK (TJ-43)	BRGHT (TJ-116)	NYT (TJ-108)	BRGHT (TJ-116) with NYT (TJ-108)
JP Ginseng	4.0 g	3.0 g	3.0 g	6.0
JP Atractylodes lancea rhizome	4.0 g	4.0 g		4.0
JP Atractylodes rhizome	—	—	4.0 g	4.0
JP Citrus unshiu peel	2.0 g	3.0 g	2.0 g	5.0
JP Poria sclerotium	4.0 g	5.0 g	4.0 g	9.0
JP Ginger	0.5 g	1.0 g	—	1.0
JP Pinellia tuber	4.0 g	6.0 g		6.0
JP *Glycyrrhiza*	1.0 g	—	1.0 g	1.0
JP Jujube	2.0 g	—	—	—
JP Magnolia bark	—	4.0 g	—	4.0
JP Perilla herb	—	2.0 g	—	2.0
JP Immature orange	—	1.5 g	—	1.5
JP Japanese angelica root	—	—	1.5 g	1.5
JP *Astragalus* root	—	—	4.0 g	4.0
JP Rehmannia root	—	—	4.0 g	4.0
JP Cinnamon bark	—	—	2.5 g	2.5
JP Polygala root	—	—	2.0 g	2.0
JP Peony root	—	—	2.0 g	2.0
JP Schisandra fruit	—	—	1.0 g	1.0
Symptoms and conditions of application	Weak stomach, loss of appetite, full stomach pit, fatigue, anemia, cold limbs	Depressed feelings, feeling of foreign body in the throat and esophagus	Declined constitution after recovery from disease, fatigue, malaise, anorexia, perspiration during sleep, cold limbs, and anemia	—
		Palpitation, dizziness, nausea, heartburn, decreased urine volume, anxiety neurosis, nervous gastritis, and hyperemesis gravidarum		
	Gastritis, gastric atony, gastroptosis, maldigestion, anorexia, gastric pain, and vomiting	Water brash and gastritis		

In the present case, we first prescribed RKT, but it was not efficacious. NYT was added, but the patient’s serum potassium level decreased to hypokalemic levels. RKT and NYT include licorice, which is a possible crude drug for pseudoaldosteronism. The combined use of RKT and NYT includes a daily dose of 2.0 g licorice. One of the most well-known adverse reactions to Kampo medicines is pseudoaldosteronism, caused by glycyrrhizin, which is included in several Kampo medicines (Arai et al., 2020). A report of patients with pseudoaldosteronism in Japan showed that even small amounts of licorice (less than 2.0 g) could cause pseudoaldosteronism, and older age is considered a risk factor for the disease (Yoshino et al., 2014). Table 1 shows the number of crude drugs used in RKT, BRGHT, and NYT. The combination of BRGHT and NYT includes almost all the crude RKT drugs in large amounts. In addition, crude drugs that promote gastrointestinal movement are added. However, the amount of licorice is limited to 1.0 g per day, and pseudoaldosteronism can be avoided. Thus, we switched to BRGHT and NYT for recovery from anorexia and nausea.

BRGHT, composed of nine crude drugs, is a Kampo medicine used to treat nausea and anxiety. Further, 7.5 g of TJ-116 BRGHT includes the crude drugs shown in Tables 1, 2 (STORK, http://mpdb.nibiohn.go.jp/stork/). We previously reported that BRGHT could inhibit corticotropin-releasing hormone receptor 2, dopamine receptors D2 and D3, neuropeptide Y receptor type 2, and acetylcholinesterase, which synergistically improves gastric emptying (Mogami et al., 2020). BRGHT administration also reduces the frequency of aspiration pneumonia in patients with brain damage (Takayama et al., 2021).

**TABLE 2:** Plant names and part of each ingredient described in the present study.

Ingredient in English	Plant name (Latin)	Plant part (Latin)
JP Atractylodes Lancea Rhizome	*Atractylodes lancea* De Candolle, or *Atractylodes chinensis* Koidzumi *Compositae*)	*Rhizoma*
JP Atractylodes rhizome	*Atractylodes japonica* Koidzumi ex Kitamura or *Atractylodes macrocephala* Koidzumi (*Atractylodes ovata* De Candolle) (*Compositae*)	*Rhizoma*
JP Cinnamon bark	*Cinnamomum cassia* Blume (*Lauraceae*)	*Cortex*
JP Citrus unshiu peel	*Citrus unshiu* Marcowicz, or *Citrus reticulata* Blanco (*Rutaceae*)	*Pericarpium*
JP Ginger	Zingiber officinale Roscoe (*Zingiberaceae*)	*Rhizoma*
JP Ginseng	Panax ginseng C. A. Meyer (*Panax schinseng* Nees) (Araliaceae)	*Radix*
JP *Glycyrrhiza*	*Glycyrrhiza uralensis* Fischer, or *Glycyrrhiza glabra* Linné *Leguminosae*)	*Radix*
JP Immature orange	*Citrus aurantium* Linné var. daidai Makino, *Citrus aurantium* Linné, or *Citrus natsudaidai* Hayata (*Rutaceae*)	*Fructus immaturus*
JP Japanese angelica root	*Angelica acutiloba* Kitagawa, or *Angelica acutiloba* Kitagawa var. *sugiyamae* Hikino (*Umbelliferae*)	*Radix*
JP Jujube	*Zizyphus jujuba* Miller var. *inermis* Rehder (*Rhamnaceae*)	*Fructus*
JP Magnolia bark	*Magnolia obovata* Thunberg (*Magnolia hypoleuca* Siebold et Zuccarini), *Magnolia officinalis* Rehder et Wilson, or *Magnolia officinalis* Rehder et Wilson var. *biloba* Rehder et Wilson (*Magnoliaceae*)	*Cortex*
JP Perilla herb	*Perilla frutescens* Britton var. *crispa* W. Deane (*Labiatae*)	*Herba*
JP Pinellia tuber	*Pinellia ternata* Breitenbach (*Araceae*)	*Tuber*
JP Polygala root	*Polygala tenuifolia* Willdenow (*Polygalaceae*)	*Radix*
JP Poria sclerotium	*Wolfiporia cocos* Ryvarden et Gilbertson (*Poria cocos* Wolf) (*Polyporaceae*)	—
JP Schisandra fruit	*Schisandra chinensis* Baillon (*Schisandraceae*)	*Fructus*

NYT is a Kampo medicine, composed of 12 crude drugs, used to recover from disease, fatigue, and anorexia. Additionally, 9.0 g of TJ-108 NYT includes the crude drugs shown in Tables 1, 2 (STORK, http://mpdb.nibiohn.go.jp/stork/). A review of RCTs of NYT demonstrated the usefulness of NYT in the treatment of cancer and related conditions (Takayama et al., 2019). Other studies have reported increased food intake *via* the activation of orexigenic OX1R-expressing neurons in the hypothalamus (Miyano et al., 2020), maintenance of nutritional status in patients with wasting conditions (Sasatani et al., 2020), and the activation of both ghrelin-responsive and ghrelin-unresponsive neuropeptide Y pathways for the treatment of anorectic conditions, which are associated with cancer or frailty (Goswami et al., 2019). These studies support the potential of NYT in cancer patients with anorexia, malnutrition, and mental disorders.

RKT is also used to treat chemotherapy-induced anorexia. The mechanisms of appetite improvement through ghrelin signaling have been reported in several studies (Asakawa et al., 2001; Nakazato et al., 2001; Takeda et al., 2008). Yoshiya et al. (2020) reported that RKT could mitigate chemotherapy-induced anorexia and ameliorate acylated ghrelin levels in the plasma, decreasing anorexia during the delayed phase of cisplatin-based chemotherapy in cancer patients.

RKT is used to improve nausea and anorexia *via* ghrelin signaling, while NYT is used to improve anorexia, malnutrition, and anxiety. BRGHT improves both upper gastrointestinal motility *via* multiple signaling (D2, D3, neuropeptide Y, and acetylcholinesterase) and anxiety. Over 60% of metastatic melanoma survivors treated with ICIs experience anxiety while waiting for test results, fear of recurrence, and death (Lai-Kwon et al., 2019). Anxiety could result in appetite loss, gastrointestinal impairment, and malnutrition. The patient in the presented case may have had anxiety because of metastatic melanoma and long-term chemotherapy. Therefore, the combination of NYT and BRGHT would be more effective for improving malnutrition, upper gastrointestinal dysfunction, and anxiety. According to the reports above and the present case, the combined use of BRGHT and NYT allows recovery from appetite loss, nausea, and malnutrition during cancer treatment.

Interstitial lung disease (ILD) or interstitial pneumonitis (IP) are important adverse events of ICIs. The occurrence rate of these adverse events is reported as 7.2% for nivolumab with ipilimumab (Opdiva, 2020). In the present case, computed tomography and blood sampling did not show the existence of ILD or IP. The ILD or IP occurrence rate with RKT has been reported to be 0% (Suzuki et al., 2020), and with NYT and BRGHT, it has never been reported. However, the other Kampo medicine has been reported as a possible cause of ILD or IP (Arai et al., 2020); hence, when using Kampo medicine together with ICIs, careful follow-up for adverse events is recommended.

We believe the improvement of anorexia and malnutrition is related to the use of Kampo medicine; however, cancer remission is also a reason for the patient’s recovery.

## Conclusion

In a patient with advanced malignant melanoma, cancer was treated using ICIs. Side effects such as anorexia and nausea with poor nutritional status were resolved using Kampo medicines, BRGHT and NYT. The clinical course of the patient in our report shows the usefulness of Kampo medicine as supportive care for symptom relief and the maintenance of nutritional and mental status during cancer treatment.

## Patient Perspective

This case report was approved by the ethics committee of the Graduate School of Medicine, Tohoku University, Sendai, Miyagi, Japan, on 7 February 2022 (protocol identification number: No. 24377).

## Data Availability

The data that support the findings of this study are available from the corresponding author upon reasonable request.
